# Definition and assessment of frailty in older patients: the surgical, anaesthesiological and oncological perspective

**DOI:** 10.3332/ecancer.2020.1105

**Published:** 2020-09-15

**Authors:** Lorenzo Dottorini, Luca Turati, Luca Mattei, Paolo Formenti

**Affiliations:** 1 Oncology Unit, Medical Sciences Department, ASST Bergamo Est, Alzano Lombardo (BG), 24022, Italy; 2 Surgical Oncology Unit, Treviglio Hospital, Treviglio (BG), 24047, Italy; 3 Department of Neurosurgery, Fondazione IRCCS Istituto Neurologico C. Besta, Milan, 20100, Italy; 4 SC Anestesia e Rianimazione, Ospedale San Paolo—Polo Universitario, ASST Santi Paolo e Carlo, Milan, 20100, Italy

**Keywords:** elderly, surgery, anaesthetist, oncologist, geriatric, assessment

## Abstract

The number of oncology, surgery and anaesthesia procedures in older patients has greatly increased in recent years due to ageing populations. Older patients are typically characterised by physical changes such as comorbidities, decline in physiological activities and cognitive impairment. All these factors, together with polypharmacological therapies, may substantially impact perioperative outcome, quality of recovery and, more in general, quality of life. A comprehensive multidisciplinary approach to perioperative care is thus needed. The assessment of frailty has a central role in the pre-operative evaluation of older patients and, with a multidisciplinary approach. The best surgical procedures and oncologic therapies can be accurately discussed in the pre- and post-operative periods. All clinicians involved in this scenario should be proactive in multidisciplinary care to achieve better outcomes.

## Introduction and background

It is well known that cancer is typically an age-related disease with approximately 60% of all newly diagnosed malignant tumours and 70% of all cancer deaths occurring in persons aged 65 years or older [1]. This older population growth raises the question of selecting those patients who will be able to tolerate surgical and anticancer treatments. In fact, it is supposed that almost 50% of patients >70 years will experience severe chemotherapy-related toxicity [2], and 60% will have post-operative complications after colorectal cancer surgery, 78% of which will be severe ones [3]. One of the most difficult things when setting up a therapeutic programme for an older patient is to estimate the risk/benefit balance of any therapeutic proposal for each of them. In fact, ageing is a highly heterogeneous process, and sometimes, the so-called ‘clinical eye’ is not able to take into account the individual quality, physiological reserves, family context and the potential risks of the planned chemotherapy treatment or surgery. A great number of tools and scales have been developed to identify the ‘frail’ patients, but a clear and universally recognised definition of frailty is still debated. With this paper, we aim to define the state of the art in surgical, anaesthesiological and oncological patients.

## The surgical point of view

The constant and progressive ageing of the population in recent years has led to an increase in the number of surgeries in older cancer patients and consequently also the risk of mortality and morbidity after such operations [4]. In the past decades, increasing evidence of scientific literature showed the importance of a comprehensive geriatric assessment (CGA) before surgery in older patients [5]. Moreover, the American College of Surgeons (ACS), the American Geriatric Society and the International Society of Geriatric Oncology (SIOG) recommend the evaluation of older patient in perioperative setting [6]. A recent study by Ghignone *et al* [7] recruited a SIOG surgical task force and made a survey on surgeons’ attitude on older patients. They found that only 6% of surgeons used CGA in pre-operative setting, only 8% asked about activity of daily living (ADL) and instrumental activity of daily living (IADL) and interaction with geriatricians was low (36%), demonstrating how in everyday clinical practice a deep assessment of older patient is still unusual. Historically, patients about to undergo surgery are evaluated with the American Society of Anaesthesiologists (ASA) score [8], but recent scientific evidence showed the limit of this evaluation and the importance of a deeper evaluation, especially in older patients. Pope *et al* [9] developed a tool called ‘Preoperative Assessment in Elderly Cancer Patients’ (PACE) which considers parameters as cognition, functional status (ADL and IADL), depression, comorbidities, ASA score, ECOG performance status and fatigue to define which patients could be at risk for surgical complications. A further SIOG surgical task force prospective study by Audisio *et al* [10] evaluated the PACE tool on 460 consecutive older patients undergoing surgery. The authors demonstrated how a decrease in parameters included in PACE tool was associated with a 50% increase in the relative risk of post-operative complications; particularly, a moderate/severe fatigue, a dependent IADL and an abnormal performance status were seen to be the most important independent predictors of post-surgical complications. Min *et al* [11] assessed the Vulnerable Elders Surgical Pathways and Outcomes Assessment (VESPA) tool in pre-operative setting. VESPA tool included the evaluation of six basic activities of daily living items (bathing, dressing, transferring, feeding, grooming and toileting), eight instrumental activities of daily living items (medication administration, meal preparation, telephone use, transportation, shopping, housekeeping, laundry and finances), gait speed, mobility, number of fall in the past year and the Timed Up and Go (TUG) test and also introduced a new item in literature with the question made to the patient: ‘Can you manage by yourself for several hours alone after your procedure (outpatient)/after discharge (inpatient)?’. They evaluated a total of 711 patients and found out that the number of difficulties with the activities of daily living, anticipated difficulty with postoperative self-care, Charlson comorbidity score of 2 or more versus less than 2, male sex and work-related relative value units were independently associated with postoperative complications. The conclusion of the authors is that a pre-operative assessment could be performed by non-surgeon healthcare personnel and could help to identify patients at a higher risk of post-operative complications. A further prospective study by Pollock *et al* [12] evaluated 476 patients undergoing oncologic surgery with VESPA tool, finding that patients with high VESPA score (≥9) had a longer length of stay in hospital (6.6 versus 2 days), more geriatric complications (39.5% versus 5.7%), more surgical complications (29.5% versus 11.8%) and more post-discharge needs confirming the potential utility of this tool in pre-operative setting. Montroni *et al* [13] proposed a therapeutic algorithm for the management of elderly patients with rectal cancer identifying in G8 score (a screening test for vulnerability in older patients), Mini-Cog score, TUG test and history of falls, the most important factors predicting a possible frailty and suggesting that patients with a deficiency in one of these tests underwent a more in-depth geriatric evaluation to decide whether the surgical treatment should, therefore, be personalised or standard. The same authors subsequently investigated the Geriatric Oncology Surgical Assessment and Functional rEcovery [14]. The trial prospectively evaluated, with a 30- and 90-day follow-up, 417 patients >70 years undergoing surgery with a baseline complete geriatric assessment. The study showed that a complete pre-surgical geriatric evaluation is possible in everyday clinical practice and that a condition of frailty is very often present in pre-surgical assessment, and an interim analysis showed the possible impact of assessing surgical outcomes and quality of life of those patients; further follow-up will better clarify the role of geriatric assessment on surgery. The American College of Surgeons National Surgical Quality Improvement Programme (ACS NSQIP) developed a risk calculator validated on 1,414,006 patients with the goal to provide accurate, patient-specific risk information to guide both surgical decision-making and informed consent; ACS NSQIP uses 20 patient predictors and the planned procedure to predict the chance that patients will have any of 18 defined outcomes within 30 days following surgery [15].

## The anaesthesiological point of view

Older patients are at an increased risk of surgical complications, and this makes pre-operative evaluation by an anaesthesiologist more difficult [16]. As mentioned above, the ASA score is one of the most commonly recorded physical classification systems [17]. Many studies indicate the relationship between the ASA score and morbidity, mortality, hospitalisation, postoperative infections and healthcare costs in both pre- and post-operative settings [18, 19]. Despite its wide use, the ASA score has some limitations: first, it is a subjective assessment. In fact, it is not uncommon that two anaesthetists differ in the ASA score when they assign to a particular patient. Second, the ASA score seems not to be enough to fully evaluate older patients. Increasing scientific evidence suggests that elements from a geriatric assessment, such as functional status, comorbidity, cognitive function, nutritional status and depression, are more sensitive and specific predictors of surgical outcomes in older patients with cancer [9, 10, 20, 21]. The objective measurements of physical functions have been tested in pre-operative setting: the slow gait speed is a simple test assessing the time for a patient to walk 5 m in > 6 seconds, and its use in this setting has been associated with a higher risk of mortality and morbidity after cardiac surgery [22]. For instance, the cardiopulmonary exercise testing is a specific evaluation of cardiorespiratory reserve able to simulate the levels of physical stress to which a patient is subjected during surgery [23]. It allows identifying cardiopulmonary comorbidities never highlighted before the test, surely incremented in older patients. Impaired functional capacity and reduced cardiopulmonary function have been associated with all-cause mortality after major elective intra-abdominal surgery, complications and hospital length of stay [24, 25]. The assessment of nutritional status in older patients undergoing surgery seems to play a key role in clinical outcome [26]. The most important factors to evaluate the nutritional status of patients are significant weight loss, low body mass index, hypo-albuminemia and significant and sustained reduction in food intake [27, 28]. The causes can be related to functional decline, presence of comorbidities, increased symptom burden, psychosocial determinants and anticancer treatments. All these causes can lead to a global anorexia–cachexia syndrome which is seen in approximately 50% of cancer patients and is responsible for increased post-operative complications, mortality and increased length of hospitalisation [29, 30]. Even the evaluation of the home therapy must be accurate, it is well known that pharmacokinetics in older patients is influenced by physiological reduction of renal function [31] and is not unusual to find, in older patients, an increased proportion of body fat with a decreased proportion of body water with a consequent modification of the volume distribution for lipid-soluble and water-soluble drugs [32]. In addition, we have to consider also the possible use of anticancer or major analgesic therapies, and this could lead to dangerous interactions with anaesthesiological drugs [33, 34]. When focusing on specific anaesthesiological drugs, it is important to notice that the minimum alveolar concentration of all inhalation agents decreases with age to values up to 30% lower in 80 years’ patients so that the concentrations of drugs must be lower for a given depth of anaesthesia [35]. The dosing of induction agents such as thiopental must be well verified because it is well known that the sleep-dose requirement of thiopental decreases with age, possibly due to changes in pharmacokinetics rather than pharmacodynamics [36, 37]. This interaction could lead to a prolonged awakening time. In general, we can state that the agent’s induction dose is lower in older patients and the induction/awakening times are pro-longed. Thus, a careful titration of the dose is mandatory in order to avoid hemodynamic instability and prolonged awakening time.

## The oncological point of view

The definition of frailty in geriatric oncology is very difficult, and still, there is no universal consensus about the right definition. Frailty in oncology could be defined as an impairment of at least one of the four geriatric domains evaluated with CGA [38, 39]. The CGA is an evaluation of older patient, with the purpose of planning healthcare assistance [40, 41]. It consists of a series of tests and evaluations, which can help the clinician to better understand the global health status of the patient. CGA is able to uncover problems that would not, otherwise, be identified by a routine history and can predict the risk of chemotherapy toxicity, as well as functional decline and mortality, and its use is recommended by the ASCO international guidelines [39]. One of the biggest problems of CGA is very long to administer and time consuming, so, in everyday clinical practice, it is very difficult to be applied. Some alternative screening tools, such as Geriatric 8 (G8) and Vulnerable Elderly Survey-13 (VES-13), have been proposed to select which patients could benefit from a complete evaluation with a CGA [39, 42]. G8 is an eight-item tool derived from Mini Nutritional Assessment (MNA). It includes questions related to food intake, weight loss, mobility, neuropsychological problems, body mass index, medication use, self-rated health and age [43]. Bellera *et al* [44] validated the G8 on a population of 364 elderly patients affected by different tumours undergoing a first-line chemotherapy and showed good sensitivity and specificity (85% and 65%, respectively). Clinical trials demonstrated that G8 is strongly prognostic for functional decline on ADLs/IADLs and overall survival [45]. Furthermore, an association with early mortality [46] and a potential role in predicting side effects even in non-chemotherapy treatments have been demonstrated [47]. VES-13 is a scale containing 13 questions considering age, self-assessment of health and possible difficulties in performing functional and physical activities [48]. VES-13 was validated by Luciani *et al* [49] in a study of 648 patients aged >65 years with solid or hematologic malignancies and demonstrated that patients who were vulnerable according to the VES-13 were at significantly increased risk of haematologic and non-haematologic toxicity. According to the national and international guidelines, patients with impairments in VES-13 domains should undergo a full CGA [50]. VES-13 score has been shown to be associated with mortality, chemotherapy toxicity and functional decline [51]. Even more specific tools for assessing the risk of chemotherapy toxicity were validated in the past decades: the Cancer and Ageing Research Group (CARG) score was developed by Hurria *et al* [43] and consists of a predictive model for chemotherapy toxicity; CARG score consists of 11 items including age of the patients, type of cancer (gastrointestinal or genitourinary cancer versus other), number of chemotherapeutic drugs (mono versus polychemotherapy), dosing of chemotherapy (standard versus reduced dose), laboratory factors (creatinine clearance and level of haemoglobin) and geriatric assessment variables (hearing, number of falls in the past 6 months, ability in taking medicine, ability to walk one block and social activity) [6]. CARG score was validated in patients >65 years, is freely available online, takes 5 minutes to be done and calculates the estimated risk of any grade 3 to 5 toxicity. Extermann *et al* [2] developed the chemotherapy risk assessment scale for high-age patients (CRASH) score for patients aged > 70 years. This tool considers IADL, lactate dehydrogenase value, diastolic blood pressure and published toxicity of the chemotherapy drugs as predictive for haematological toxicity. Other factors such as malnutrition (evaluated with MNA score [52]), cognition (evaluated with mini-mental status examination—MMSE score [53]) and ECOG Performance Status score [54] were the predictors of non-haematological toxicity. CRASH takes longer than CARG, usually up to 20 minutes, and it is able to evaluate the risk of haematological and non-haematological chemotherapy toxicity and is recommended by the international guidelines. Besides the evaluations already described, the other evaluations of older patients are recommended as a fundamental part of the global evaluation of the patient: the evaluation of IADL [55] is recommended, and several studies demonstrated how an impairment in any of the areas considered are associated with the risk of chemotherapy toxicity, mortality, hospitalisations and functional decline [56]. Falls are common in older patients, and its evaluation is important: a simple question ‘how many falls have you had over the last six months’ must be done before starting a treatment because falls have been associated with chemotherapy toxicity. A complete anamnestic collection is important to evaluate the number of comorbidities of the patients, which are associated with the risk of chemotherapy toxicity, poorer survival, mortality and hospitalisations. Moreover, the evaluation of depression with validated geriatric depression scale plays an important role in the evaluation of older patients; indeed, a state of depression has been associated with unexpected hospitalisations, treatments tolerance, mortality and functional decline [57]. Assessing the cognition in older patient should be a key part in geriatric evaluation: the use of validated tools such MMSE or Mini-Cog is recommended by international guidelines, and a low score in these evaluations has been associated with poorer survival and increased chemotherapy toxicity risk [39].

## Conclusions

This review was intended to highlight the current oncological, surgical and anaesthesiological knowledge concerning the elderly population. With the review of the literature, it is clear that the multidimensional geriatric evaluation represents an extremely useful and important tool in everyday clinical practice for all older patients undergoing surgery or oncologic treatments. Their careful pre-surgical evaluation and precautions even during surgery could avoid many complications during surgery and during post-operative stay in hospital, but, unfortunately, we have seen that, in real-life, there are few surgeons and surgical centres that apply this evaluation model. We hope that, in the future, older patients will gain attention from the medical class because, given the constant increase in the average age, the number of these patients will increase more and more, and willingly or not, all health professionals will have to interface with this population.

## Conflicts of interest

The authors declare that they have no conflicts of interest.

## Funding statement

No funding was received.
